# A population health approach in education to support children’s early development: A Critical Interpretive Synthesis

**DOI:** 10.1371/journal.pone.0218403

**Published:** 2019-06-14

**Authors:** Ashleigh L. Wilson, Jessie M. Jovanovic, Yasmin E. Harman-Smith, Paul R. Ward

**Affiliations:** 1 Telethon Kids Institute, University of Western Australia, Perth, Western Australia, Australia; 2 College of Medicine and Public Health, Flinders University, Bedford Park, South Australia, Australia; 3 College of Education, Psychology and Social Work, Flinders University, Bedford Park, South Australia, Australia; University of South Florida, UNITED STATES

## Abstract

The primary objective of this review is to investigate what is currently known about early childhood education planning, population health models and their relation to children’s development. A systematic review using the Critical Interpretive Synthesis method was undertaken, guided by a preliminary research question, *“How can a population heath approach be applied to educational planning to support children’s early development*?*”* which acted as a compass and guide throughout the process. The initial search yielded 20,122 results, of which 42 were included in the review. Four synthetic constructs emerged (1) Elements of population health models exist within communities and can help improve outcomes for more children, (2) Inter-disciplinary collaboration and partnerships possess unique opportunities to influence children’s development, (3) Children’s development can be influenced at a variety of levels, and (4) System change requires a range of drivers and supports. Within education, there are several models which are used to improve outcomes for children and families. Although a population health approach to planning does not explicitly exist, the results from this review indicate that it would indeed be plausible to adapt the population health approach to sites and schools, and that doing so would be advantageous for children’s development. However, implementing such an approach requires more than desire for change and demands system changes and supports. A protocol for the review was published on the International Prospective Register of Systematic Reviews (PROSPERO), registration number CRD42018098835 on 31^st^ July 2018.

## Introduction

### Children’s early development

Early childhood, defined as birth through age eight, is well recognised as a critical stage of development [1–4]. Children’s development in these years in known to have significant and lasting impacts on their later physical, social and emotional health, as well as academic achievement and employment [5–8].

Children’s growth, learning and development are influenced by a number of factors including environmental, familial, geographical and socio-economic, and can often be anticipated early in life [9, 10]. For example, academic achievement and cognitive development can be predicted by children’s exposure to socioeconomic disadvantage [11]. Education can help mediate between early life socioeconomic status and adult mortality, however, upon school entry many children have already faced significant adversity [12]. These experiences can present as challenges integrating into the classroom and without intervention children are likely to fall behind their peers as they continue through school [13]. Children who score below the 10^th^ percentile in one or more domains as described by the Australian Early Development Census (AEDC) at age five were more likely to be in the bottom 20% of students’ scores on the National Assessment Program–Literacy and Numeracy (NAPLAN) assessments at grade 3,5 and 7 [14]. These children may demonstrate a lower than average ability in one or more of the areas of basic physical health and wellbeing, social competence, emotional maturity, language and cognitive skills or communication skills and general knowledge. An absence of basic competencies in any of these areas, coupled with prior adversities can present in problematic behaviours in the classroom such as poor emotional self-regulation and difficulty interacting with peers, resulting in teachers (educators) spending more time managing their classroom and less time spent supporting learning [15, 16].

Research by McCain, Mustard [17] suggests that policies and programs that aim to reduce inequity are critical to improving outcomes for children. In addition, numerous studies show that investments in the early years are one of the most cost-efficient investments in human capital, leading to a country’s sustainable development [18, 19]. These trajectories and predictive models have driven support for intervention in the early years with the view that it will have a lasting impact on later adult health, wellbeing and academic achievement. Population health approaches are present in health, where large population level datasets are regularly relied on for tracking trends and identifying potential areas of need. An opportunity exists for educators in Australia to use this data to similarly track children’s development at a higher level.

Educators in both schools and early childhood education and care sites are being increasingly asked to consider different types of data in their planning for children’s learning and development; and while on-entry assessments and standardised testing such as NAPLAN are common practice, population level data sets such as the AEDC are often unfamiliar. Population data sets such as the AEDC are fairly new to the education system, and could help provide an insight into children’s early experiences and their communities. Adapted for use in Australia from the Canadian version of the Early Development Instrument, the AEDC is a population measure of how young children have developed by the time they enter their first year of full-time school [20, 21]. The census reports on communities, rather than individuals and can help governments, sites, schools and communities to understand the environments and experiences children are exposed to from birth to school age. Australia is currently the only country to regularly collect these data through a national census, making it an invaluable dataset that can be drawn upon for planning and to establish community partnerships while also posing unique challenges for educators who are increasingly expected to integrate the data into their planning.

To adapt to new ways of utilising data, Education might choose to look to other sectors where this has become common practice. Health is one such sector, where a common approach has been developed for using population data sets for tracking trends and identifying potential areas of need. An opportunity exists for educators in Australia to use AEDC data to similarly track trends in children’s development at a population level. The AEDC is a rich data source that could also be drawn upon in education better understand the factors driving children’s development, and subsequently inform planning to address underlying factors influencing the learning needs of children in their community.

### Construct definitions

Due to the interdisciplinary nature of this work it is important to define some key terms which have been used throughout the paper, to ensure inclusivity of the birth to eight sector, health and education.

Educators—Inclusive of all staff involved in teaching and learning duties in prior-to-school and early years of school sites [22, 23]

Leaders—School principals, early childhood education and care directors and staff involved in educational policy roles such as partnership coordinators.

Learning—“A natural process of exploration that children engage in from birth as they expand their intellectual, physical, social, emotional and creative capacities. Early learning is closely linked to early development” [22]

Development—“Knowledge of age-related characteristics that permits general predictions about what experiences are likely to best promote children’s learning and development” [24]

Population data—Data that is not available at the individual level, but is instead aggregated for groups.

### Population health approach to planning

The population health approach is becoming increasingly recognised for reducing healthcare demand and contributing to health system sustainability [25]. Despite a lack of an official definition, the population health approach aims to improve the health of entire populations and reduce health inequities among population groups by considering the risk factors and conditions that influence health [26]. Additional key elements and actions that can be used to characterise a population health approach include: a focus on the health of populations, addressing the determinants of health and their interactions, basing decisions on evidence, applying multiple strategies, employing mechanisms for public involvement, collaborating across sectors and levels, increasing upstream investments and demonstrating accountability for health outcomes [26–29] Early childhood educators, in both prior-to-school and school settings, already apply some of these concepts in their work. This paper seeks to draw comparisons between the ways in which education and health use data to inform their planning and the extent to which lessons from a population health approach could be applied to support education to incorporate new population data sets in their planning. In Table 1 below, the key elements of a population health approach have been listed, alongside our interpretation of how these concepts may be applied in both health and education sectors. The descriptions for the health sector have been based on our interpretation of the literature, as well as the table presented by Health Canada [26, 28] on ‘key actions’ and may help to develop a shared understanding between sectors.

**Table 1:** Alignment between population health approach elements, in health and education.

Population Health Concepts	Health	Education
*Focus on*:	The health of populations using indicators for measuring health status	Children’s developmental and learning progress.
*Address the determinants of*:	Health and their interactions by analysing and measuring their relationships	Children’s progress by exploring the contextual and operational factors at play.
*Base decisions on evidence*	Emphasis on the robustness of evidence, often using randomised control trials; and drawing on a variety of data and methods throughout all stages of policy and program development, before disseminating findings.	Uses evidence/outcomes-based and descriptive studies to make decisions about educational goals and improvements for learning communities.
*Increase upstream investments*	Concerned with impact of interventions on health outcomes. Criteria is applied to select priories for investment. There is a balance of short and long term investments, and an aim to influence investments in other sectors	Concerned with impact of studies to inform the direction of, and to improve educational outcomes. Investments are both short and long term
*Apply multiple strategies*	Taking action on the determinants of health and their interactions to reduce inequities between population groups. Interventions are integrated and improve health over the lifespan. Approaches are often across multiple settings and layers Across layers of health (primary, secondary, tertiary)	Applying concepts from other disciplines, such as health and wellbeing, to education settings. Strategies tend to be singular and can be employed across the whole school or targeted to those facing challenges.
*Collaborate*:	Across sectors and levels with partners who share values and vision early in the process, with a focus on visible results. Leadership, accountability and rewards are shared.	Occurs with other educators, leaders and community partnerships.
*Employ mechanisms for public movement*:	To capture the public’s interest and contribute to health literacy	To promote family and community engagement and the value of education.
*Demonstrate accountability*:	For health outcomes through a results-based accountability framework. Measures and targets are set to demonstrate improvement, and evaluation processes put in place	For education outcomes to ensure they are evidence-informed over time; and include all involved in the learning community, as a part of a process of continuing quality improvement and reflexive practice.

By applying concepts from a population health approach to the education site, educators and leaders (principals and site directors) could leverage the diversity of aptitudes and influence of their transition partners (those who also influence children either before or during their time in school including; family, early childhood education and care service staff, education providers, community organisations and key community individuals) to mitigate risks and develop solutions aimed at improving children’s development. This would also help to promote true collaboration between prior-to-school and school settings.

### Supporting developmental trajectories

Children’s development and early education is internationally recognised as a significant contributing factor to health. The United Nations has formally recognised this importance through the Sustainable Development Goals. The fourth goal ‘ensuring inclusive and equitable quality education and promote lifelong learning opportunities for all’ specifically seeks to promote access for all children to quality education [30, 31]. These goals are also reflected in the Convention on the Rights of the Child, which stipulated the right of children to: be afforded opportunities to maximally develop their capabilities, the right to education and to develop their personality and talents through education [32].

Despite the clear impact that the first few years has on a child’s trajectory there has been little investigation of the extent to which early childhood experiences are considered and planned for in early childhood education. Additionally, planning for children using population level data and approaches is a relatively new practice within the early childhood education sector, with aggregated population data about children’s early development emerging only in the last decade or so. Moreover, little is known about the applicability of aggregated early child development data and processes for its integration into planning, posing questions for educators, leaders and their communities.

## Aims

The primary objective of this review is to investigate what is currently known about the applicability of population health approaches to planning in prior-to-school and school settings, and the extent to which applying relevant concepts such as collaboration, data use, and the consideration of risk and protective factors are likely to improve children’s outcomes. The results from this review will form the basis of a larger research project, investigating how a population health approach can be applied to educational planning to support children’s early development.

There are large amounts of literature on intradisciplinary teams within health systems, however as the focus of this review is early childhood education these will not be reviewed in detail and will only be referred to where appropriate.

## Method

### Search method

An initial scoping search using the PICO process was undertaken to identify key words and phrases that would be integral to the success of the search strategy [33, 34]. This scoping search assisted in identifying appropriate parameters, developing the exclusion and inclusion criteria, determining quality filters and refining the scope of the review. A protocol for the review was published on the International Prospective Register of Systematic Reviews (PROSPERO), registration number CRD42018098835 on 31^st^ July 2018.

After significant review, the critical interpretative synthesis (CIS) method was determined as most suitable as it enables researchers to synthesise a diverse body of evidence and enables the generation of theory with strong explanatory power [35]. As population health approaches are common in the health sector, the CIS method allowed the researchers to gather information from a wide range of interdisciplinary research and resources, while still assessing its suitability for integration into the education system. The systematic nature of the review also aims to minimise bias through the use of explicit, systematic methods and transparent explanations and analysis, providing rigour, reliability and validity to the results [36, 37].

A systematic review using the CIS method was undertaken, guided by a preliminary research question, *“How can a population heath approach be applied to educational planning to support children’s early development*?*”* which acted as a compass and guide throughout the process. Primary and secondary outcomes were also determined as a way of adding rigour to the review [38]. *“How is children’s holistic development supported throughout the early years*?*”* was identified as the primary outcome. Secondary outcomes were identified as:

- Do educators employ aspects of population health approaches in routine educational planning?
- How is data used within prior-to-school and school settings to plan educational programs?
- How do prior-to-school education and care services and schools work with their communities to ensure they are more effectively supporting the children and family’s needs?

Once the guiding research question and outcomes were identified the systematic literature search was initiated.

Keywords associated with the population health approach, social determinants of health, schools and early childhood education and care, planning and development were established and combined into a search strategy. The search was translated for relevant databases, as determined by the research team and with the assistance of a library liaison. The search strategy was constructed to return results related to the way schools can use data within their planning, rather than current educational quality and curriculum planning documents. Databases searched included: ProQuest, Medline, Emcare, Scopus and Open Grey. An example of the search strategy, as adapted for use in Proquest can be found below.

noft("population health approach" OR "public health approach" OR "population health model" OR "public health model" OR "integrated service approach" OR interdisciplinary approach OR “critical population health” OR healthy cities OR healthy communit* OR “health in all policies” OR HiALP) AND noft(school* OR "early learning centre" OR ECEC OR preschool OR child*) AND noft(wellbeing OR well-being OR develop* OR leadership OR planning*) AND stype.exact("Conference Papers & Proceedings" OR "Government & Official Publications" OR "Reports" OR "Books" OR "Scholarly Journals" OR "Dissertations & Theses") AND la.exact("English").

Table 2 displays the number of articles retrieved from each database as of the 13^th^ May 2018. Due to the potential for smaller case studies and reports of sites and schools using population health approaches a wide range of document types were included in the search. English documents: including annual reports, articles, books, case studies, commentaries, dissertations/theses, literature reviews, reports and technical reports were selected.

**Table 2:** Electronic database search results.

Database	Articles retrieved
ProQuest	13,173
Medline	450
EmCare	3446
Scopus	3032
Open grey	21
TOTAL	20,122

### Search outcome

**Fig 1:**
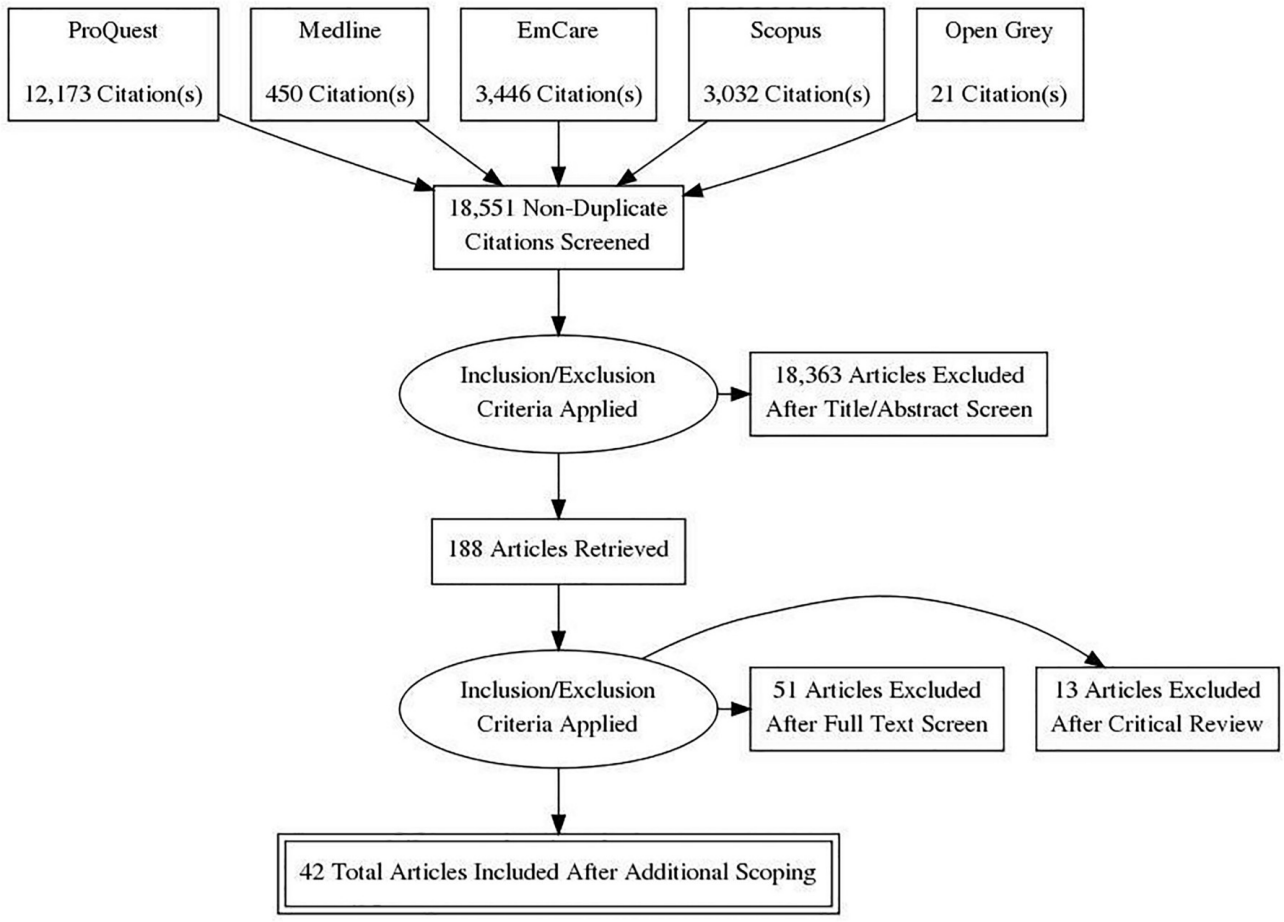
PRISMA diagram outlining the process of electronic database and other searching.

The Preferred Reporting Items for Systematic Reviews and Meta-Analyses (PRISMA) flow diagram in Fig 1 summarises the process of article selection. The initial search yielded 20,122 results, 1571 of which were identified as duplicates and removed immediately. PRISMA diagrams were used throughout the search to document the process and findings.

The inclusion criteria included any documents discussing multi-disciplinary collaboration and planning to support children’s development, public/population health models and examples of sites and schools using data. Exclusion criteria included: university or council-based programs with no collaboration with schools, interdisciplinary curriculum planning within the site or school (including assessment planning), interventions for special groups only, papers not available in English and those without any reported outcomes or recommendations. Studies identified through the literature search were uploaded to a reference manager program, EndNote X7. Study titles, abstracts and full-text were reviewed by a single researcher (AW).

### Quality appraisal

After applying the exclusion criteria, 51 articles remained. Methodological quality was based on a critical appraisal using the Joanna Briggs Institute checklists for analytical cross-sectional studies, cohort studies, qualitative research, quasi-experimental studies, systematic reviews and text & opinion pieces [35]. The types of articles included in the review were: analytical cross-sectional, cohort, qualitative, quasi-experimental, systematic reviews and text & opinion. The appraisal was undertaken independently by two researchers (AW and PW) to ensure rigor and reliability. Disagreements were resolved through discussion between the two researchers. Four articles were identified as methodologically weak, though remained in the analyses as they provided theoretical or practical insight. Thirteen papers were excluded based on the critical appraisal based on their methodological weaknesses and that they provided little or no additional insights over and above the already included papers.

The CIS approach resulted in an analysis that was iterative, interactive, dynamic and recursive [35]. This resulted in the addition and removal of articles throughout the analysis process. The reference lists of the five most relevant papers were interrogated during the appraisal process to identify any additional research. From this, four additional papers were identified and added for review.

### Data extraction

A data extraction form was developed, and extraction performed by the primary researcher (AW). Data were entered on an EXCEL spreadsheet and included the type of paper, methods, summary, key findings and concepts. The data extraction table can be found under supporting documentation 1 (S1). Each concept consisted of one to four key words or phrases that summarised the underlying themes of each article. Twenty-nine individual concepts were identified through data extraction, making up a total of five general themes once consolidated. These themes were later developed into the synthetic constructs required for a CIS.

### Synthesis

The CIS approach involves the development of synthetic constructs, which interpret and transform the evidence into a new conceptual form before developing a synthesising argument [35]. A data extraction table was used to summarise the key findings from each article and relation to the initial primary outcome. Throughout the analysis, papers were summarised but also critiqued and interrogated, with authors assumptions and biases questioned. As such, the general themes identified in initial coding were re-examined to determine four synthetic constructs. While developing the synthetic constructs, the synthesising argument also evolved.

## Results

### Development of synthetic constructs and synthesising arguments

Four synthetic constructs emerged from this CIS: (1) Elements of population health models exist within communities and can help improve outcomes for more children, (2) Inter-disciplinary collaboration and partnerships possess unique opportunities to influence children’s development, (3) Children’s development can be influenced at a variety of levels, and (4) System change requires a range of drivers and supports.

It is important to note that the constructs presented are of a theoretical nature, emerging from the researchers’ professional discourse in education, health and epidemiology. Consideration should also be paid to the key strengths of this approach, in its ability to allow the author to critique and generate theory from a wide range of evidence.

#### 1. Elements of population health approaches exist within communities and can help improve outcomes for more children

The review demonstrated that models such as Health Promoting Schools, the Whole School, Whole Community, Whole Child Approach (WSCC), and Healthy Communities are in place within schools across the world, each of which possess key elements of population health approaches. A review of how these approaches are working, could be used to identify the impact of applying such a model of planning within education. These models typically embody a focus on the health of children, their development and educational attainment, and thus reflect a commonly applied approach to population health. These approaches attempt to promote health behaviours within the school, but fail to address the underlying mechanisms, use evidence-based practices, collaborate across sectors and levels or apply multiple strategies to reduce inequities. Despite their popularity in countries such as the United States, Canada and the United Kingdom, there is currently insufficient evidence of the success of models such as these in improving children’s outcomes.

The health promoting schools’ approach for example, contains some but not all concepts from a population health approach. Although no strict definition exists, a health promoting school “constantly strengthens its capacity as a healthy setting for living, learning and working,” with a focus on: creating conditions that are conducive to health, building capacities, preventing leading causes of death, influencing health-related behaviours, making healthy decisions and caring for oneself and others [39]. The concepts applied are limited to a focus on the health of populations. Therefore, it could be argued that health promoting schools applied health education in a school setting, rather than applying population health planning methodologies within education [39]. Grants are offered by state health departments to support the increasing emphasis on health promotion in schools, and can assist with the development and implementation of programs. While these studies have clearly demonstrated the benefit of health interventions on health outcomes, and that these can be delivered in an education setting, there is little evidence that these benefits extend to other domains [39]. In education, interventions are typically implemented to improve the capacity of children to benefit from education opportunities (i.e., to improve educational outcomes by addressing factors impacting on children’s health and wellbeing). A Cochrane systematic review of the impacts of Health Promoting Schools found that of 67 eligible trials, 11 reported the impact on educational outcomes, of which only six reported on student measures [39]. Of these studies, the most commonly reported educational outcome was absenteeism, which saw a slight improvement from interventions focused on multiple risk behaviours and hand hygiene [39]. Improvements stemming from interventions on multiple risk behaviours, specifically targeting academics, character and student behaviour included: decreased student disaffection with learning, teachers’ ratings of academic motivation, improved standardised test scores for reading and maths, and reduced suspensions [39]. Additionally, programs aimed at mental health and anti-bullying also reported increased school attachment and wellbeing [39]. Despite some instances of educational improvement, the review concluded that on the whole, there was a distinct lack of evidence regarding the educational impact of the Health Promoting Schools framework [39]. With schools facing an increasing demand to support children to have good academic outcomes it could, therefore, be argued that it is unreasonable to expect schools to employ such approaches without the benefit of flow on effects to academic achievement. However, despite little empirical evidence, there are still many communities across the world employing these models within their schools. In an environment where resources and funding are typically limited and where the focus is on improving educational achievement, it is crucial that programs employed are grounded in evidence and able to demonstrate improved educational outcomes for children.

When successfully applied, elements of a population health approach such as: collaboration, inclusion, healthy environments, engagement and evidence-based practices that are embedded in policies, practice and relationships, can support children’s development [40–44]. Three of these elements also help comprise a population health approach in the form of inter-sectorial action and partnerships (collaboration), addressing the social determinants (evidence-based practices) and understanding needs and solutions through community outreach (engagement) [25]. By integrating these elements into the functioning of a site or school the potential to improve outcomes for a wider range of children is increased. Although the focus and success of these programs has typically been linked to physical health, similar principles could theoretically be applied to improve the social, emotional and cognitive development of the child. As educational practice occurs in partnership with families and communities, it is likely that children’s learning and development could be further supported if these elements of collaboration and engagement were applied outside of the school and site.

#### 2. Inter-disciplinary collaboration and partnerships possess unique opportunities to influence children’s development

As described in Table 1, collaboration, basing decisions on evidence, drawing on a variety of data and demonstrating accountability are key elements of a population health approach. Although collaboration across multiple sectors is common in public health departments, this review found that within education, inter-disciplinary collaboration appears to exist at a relatively superficial level with schools, occasionally involving communities coming together to form genuine partnerships [25, 45–47].

Where relationships were formed, data was used to support initial conversations between staff and across sectors and build accountability systems [40, 48]. Murray described how schools and jurisdictions are held accountable for academic outcomes and process measures, and how “incorporating metrics related to health and wellness into data tracking and school accountability systems (provides) educators, policy makers and the public with a refined understanding of how to achieve learning and academic outcomes” [40]. Aligning data such as attendance, discipline, behaviour and absenteeism with intervention efforts allowed schools to demonstrate the effect of their programs and inform policy, processes and practices [40]. In Belansky’s Adapted version of Intervention Mapping [48]school-level data was introduced alongside handouts of best practices to supplement conversations on what could be changed about the school environment [48]. This created a common ground where all those involved could discuss children’s development and begin their journey grounded in evidence. By working together towards a shared goal, stakeholders reduced the burden placed on any one organisation by sharing resources, contacts, knowledge and experiences [49–51]. The most common barriers to continued partnerships and programs were time and money [52, 53]. However, where groups pooled their resources these barriers were reduced often leading to sustained programs and improved outcomes [51]. Additionally, sharing knowledge of the community and their challenges and strengths allowed for more suitable and ultimately successful response [54].

A focus on building genuine relationships, and a commonality of intent and shared goals were key components of success [55]. Additional elements for successful partnerships included: active and engaged leadership, effective use of data, integration of the process within the existing site and school improvement process, distributed team leadership, ongoing and embedded professional development and creation or modification of policy [56]. In one such example, Toronto First Duty (TFD) brought together kindergarten, child care and parenting supports into a single program and produced positive outcomes for children’s development as well as improved quality of family life [57]. TFD demonstrated short-term positive effects on children’s social-emotional development on the Early Development Instrument and found that more intense use of the program (i.e. higher dose) also predicted children’s cognitive and language development [57]. Elements from this prior-to-school approach could be applied to school settings to develop a successful collaborative model.

The results of the search returned minimal results demonstrating how schools work with other stakeholders in the community to support children’s development in the years prior to them beginning their formal education. This is likely due to a lack of time, funding and support for this type of research, with education research focused on educational constructs (e.g. curriculum, pedagogy, educational leadership). This does not mean that schools were not engaging with community. Community engagement is a recognised strategy for enabling schools to best facilitate learning and improve their educational outcomes for children [58]. Accounting for constraints around time, funding and support, schools may simply not envisage their work as having broader health- or child development-focussed outcomes. Nevertheless, there has been a call for schools to play a larger leadership role, allowing them to address health in a more strategic manner; supporting the idea that schools could have a more active role in communities [59]. As the factors influencing learning and development are complex and multifaceted, it is important to consider the role of the school in children’s lives and their ability to bridge critical home and community ecologies. Regardless of their position in the community, either leadership or an active member of a local partnership, schools will continue to be a universal access point for families, creating a natural hub and the ability to play a significant role in the development of children in the community. It could, therefore, be argued that more clarity around the shared intention of improved educational and life outcomes for children is required before health and education sectors ‘buy in’ to the use of population-level data in their respective and shared professional practices [60–62].

#### 3. Children’s development can be influenced at a variety of levels

The review demonstrated that the impact of the family and community on children’s development is well known and is often the focus of health campaigns. Like the population health model, the findings of this study suggest that education sectors are beginning to move towards a holistic approach and are considering the impact of the family and the community on development. However, an opportunity exists for sites and schools to further draw on these influencers to support children’s development both before they reach school and once formal education has begun. Initial search results revealed a large number of articles outlining how planning occurs for children’s needs at the individual level, including Individual Learning Plans which are used by schools across Australia [63]. There remains scope to increase the extent to which planning for children in the community is based on their holistic needs. By shifting the focus away from the individual, educators can anticipate the needs of their incoming cohorts and work to improve outcomes before they enter the classroom, working towards more upstream intervention strategies. For the individual, educators can use this knowledge to better understand how their learners’ needs are shaped by a range of sociological factors. In turn, these considerations also help schools consider where strategic partnerships may be required to overcome systematic barriers such as waiting lists for assessment of support needs.

The research reviewed, indicated that population health approaches that focus on strengthening protective factors in families and promoting the development of children are able to improve children’s outcomes [64]. Reminiscent of the theory of proportional universality, common to the public health approach, a mixture of targeted high-intensity services for those requiring additional support and universal services for all that address risk and protective factors, addressing the determinants of health and their interactions, have been found to enhance children’s developmental trajectories [51]. Currently, school-based universal interventions are commonly focused on addressing the needs of educators and the site by improving school structure, supporting educator’s pedagogy and instructional policy, rather than focusing on the needs of the children [65]. Population health approaches argue that focusing only on children with complex needs fails to address the needs of all children and thereby limits its potential for widespread impact in a community [66]. Additionally, the prevention paradox understands that multiple levels of intervention are required to prevent poor outcomes in childhood and adolescence. A mix of universal interventions, selective interventions focusing on at-risk groups and indicated interventions for those already facing challenges are required [65]. By addressing risk at multiple levels, those who are at the highest risk are able to receive targeted support, while those who would typically be overlooked due to a lack of risk factors are also identified. However, in order to reach such a large group, cross-sector collaboration and policy that enables collaboration is essential.

Finally, the review highlighted that interventions that focus efforts on a single program or intervention are unlikely to create sustained improvement and instead, that systems need to change to support children and families, and that this type of change is driven by changes to organisational policy [66]. Generation of political support and building policy that promotes positive factors for children’s learning and development, could be considered another key element of a population health approach [26]. The review demonstrated that investment by local authorities can provide much needed support to sites and schools, and can have a significant impact on priorities, enhancing health and supporting academic achievement [67]. Policy in an educational setting that promotes the healthy development of all children can take many forms including: promoting integrated systems of care, ensuring optimal use of existing resources and enabling the use of data to document issues and inform advocacy [26, 66]. In summary, improvements require an individual acting as a champion, data to evidence the needs (of the local community) and educational policies and procedures that have sufficient flexibility to reflexive change to be enacted.

#### 4. System change requires a range of drivers and supports

This review highlighted the importance of considering how population change (improvement for many children in a community) is achieved and maintained. In order to achieve sustainable change, supports are required at the leadership level. Where schools have been successful in implementing population health models support from senior staff was essential [50, 68]. Additionally, programs where there was a staff member acting as a champion for the community or intervention were also more likely to have success [69]. Described as a key action for collaborating across sectors and levels, identifying and supporting a champion also occurs in a population health approach. Rooney [68] describes the Whole School, Whole Community, Whole Child Model and the importance of having a strong leader who can advocate, communicate and coordinate throughout the process. Leadership also supported the success of the program by clearly linking the model initiatives to academic indicators establishing buy-in and sustainability [68]. Conversely, where there was little or no support from leadership or a key champion was no longer in the role, the project was more likely to fail [47].

A clear link to existing work such as curriculum planning and reporting may be useful to promote a population health approach. Educators are already burdened with large amounts of paperwork and duties, therefore building on or modifying existing systems rather than adding new activities or programs is often a more successful approach [70]. Given the existing requirements within education to: engage in site and quality improvement planning, document how these plans are enacted, engage in collaborative work and use evidence to support planning, there remains an opportunity to improve children’s learning and outcomes by drawing on the systems already in place.

The review highlighted the potential to draw on the theory of diffusion of innovations (DOI). The DOI theory seeks to explain the how, why and the rate at which new ideas spread [71]. Rogers theorises that four main elements influence the spread of an idea: 1. The innovation itself, 2. Communication channels, 3. Time, 4. A social system. Each of these elements are made up of adopters characterised as innovators, early adopters, early majority, late majority and lagers, each with different motivations and requirements for taking on a new idea [71]. Drawing on this theory, schools could be supported to adopt aspects of a population health approach within their planning, by identifying and tapping into existing supports within the education system. DOI theory can also be used as a reflective tool to review change programs and identify emerging barriers to successful implementation [72]. For some, proven stories of success are required before implementing change. Academic partnerships were discussed as being able to provide such stories and therefore support the development an uptake of new programs [48]. When implementing a new approach, it will be important to consider both the target population and those executing the changes to ensure appropriate supports and motivators are in place.

## Discussion

This review has identified four synthetic constructs that attempt to interpret some of the research in the area of educational planning, in order to respond to the original question: “*How can a population heath approach be applied to educational planning to support children’s early development*?*”* The four constructs identified from the literature were: elements of population health approaches exist within education communities and can help improve outcomes for more children; inter-disciplinary collaboration and partnerships possess unique opportunities to influence children’s development; children’s development can be influenced at a variety of levels, and system change requires a range of drivers and supports. Despite their differing sources, these concepts contain unifying themes that can be used to draw generalisations from the findings.

Several elements of a population health approach were identified within the education system including focusing on the health (educational attainment) of children, addressing the determinants of health and their interactions, basing decisions on evidence, collaboration across sectors and levels, and identification of a key champion. Though these elements are present in various programs and processes, there was no evidence of all eight approaches being employed simultaneously. Population health approach elements that were not demonstrated in the review included: increase upstream investments, employ mechanisms for public involvement, demonstrate accountability for health systems. Therefore, it could be argued that although the education sector does draw on elements of a population health approach, they are often applying health or wellbeing interventions and are not employing the complete approach, missing out on a critical opportunity to maximise return from their efforts and improve outcomes for children.

There appears to be two distinct areas of opportunity to integrate population health approaches into planning. The first, would be to include it in the work that is already taking place in prior-to-school and school settings through planning for children’s needs based on their past experiences. Continuity of learning and successful transitions have been argued to play a crucial role in children’s education success and their ability to maximise learning opportunities [73]. As such, there is a requirement within education systems to ensure programs are organised in ways that maximise opportunities for each child’s learning [74]. Educators who are prepared for their cohorts are better placed to support children at crucial transitions, thereby increasing the proportion of children who experience continuity in their learning rather than disruption. This review has identified the potential benefit of incorporating information about the factors influencing children’s development into educational planning to better encompass and build on children’s previous experiences and to anticipate how these may continue to impact their learning, development and capacity to engage with learning opportunities. Importantly, properly designed and managed education programs have been shown to generate large returns on investment, primarily in the way of savings in relation to reducing conditions later in life [75]. Several models have been proposed to explain the effect of socioeconomic status and ultimately life experiences on later life outcomes and each provides an argument for education systems to play a substantial role in reducing upstream burdens for individuals, communities and economies. The timing model suggests that socioeconomic factors have the greatest influence if experienced during specific developmental periods such as birth to three years [76]. Within education, research suggests that systems need to respond early in children’s lives and provides an impetus for schools to advocate for children before they enter formal education, in order to avoid challenges later in life. Conversely, the accumulation model suggests that the detrimental effects of socioeconomic status can accumulate throughout the life course and will continue to do so with increasing duration of exposure to disadvantage [76]. This model may help educators consider the factors that have influenced children’s development and consider how these may present the child with additional challenges and barriers to engagement in learning. Although prior-to-school and school settings are not able to impact socioeconomic status (SES), there are instances where education has also been able to help mediate some of the negative impacts of SES [12]. Regardless of their differences, these models each emphasise the importance of early sensitive periods and their impact on later health and development. If schools have insight into the capabilities children bring with them to school and plan an education experiences that is well placed to build on these capabilities, there is not only a greater opportunity to cater to children’s needs but also an increased likelihood of developmental gains.

The second area of opportunity to integrate a population health approach, would be to build on what is already occurring and increase the outreach into the community. The significance of partnerships emerged as a recurring theme across the four constructs. Partnerships with community stakeholders and families supported children’s development by reducing inequities in access, ensuring all children were connected with the school, and children were receiving the services and supports they required. Once children are enrolled in school there are many programs and interventions available to support children and families and improve health. However, there appears to be multiple challenges facing schools and their ability to increase their reach into the community prior to children starting school including funding, role constraints and data sharing [53]. To overcome these challenges, schools could employ aspects of a population health approach to working together with other stakeholders in the community, to promote healthy child development before school entry. This could support families and the community, so that their children can arrive at school with an increased capacity to learn. Early intervention programs can have positive effects on children’s developmental trajectories and learning, particularly when applied prior to school age [77, 78]. Local approaches that focus on addressing risk factors and promoting protective factors at a community level are not only more cost efficient but could also improve the success of the program by ensuring it reaches all children during the crucial years of development [79, 80].

Data was discussed throughout the literature and across the constructs as supporting planning to ensure actions were grounded in evidence. Despite the recurring references to its importance there was a lack of discussion about how to use data at a population level, with the majority of use centring around individual children. Despite the known interactions between early childhood education and later life outcomes it appears as though there is a wariness of educational research and practice towards health paradigms. This may be due to educators feeling as though they do not possess the skills to draw these connections or being unable to obtain the appropriate data required to draw such links [81]. Within Australia, large datasets such as the AEDC can be utilised to demonstrate the connections between education and later life outcomes. Demonstrating these links can support diverse sectors to form partnerships to address shared concerns. Through interrogating the AEDC data educators and education leaders can identify and understand where children in the community are facing challenges and explore what may have impacted their development. Becoming more aware of children’s contexts and early experiences supports sites and schools to be prepared for their incoming cohorts. Trend data, such as that from the AEDC can help to identify where there are protective and risk factors at a community, state or national level, and help educators to consider appropriate resources that can help them address the needs of incoming cohorts. In turn, educators are likely to be better placed to develop a suitable curriculum and by understanding the source of the problems can put in place supports for children to reduce the time spent reacting to the everyday problems presented in the classroom. Further support in the way of professional development or integration into early childhood education courses, may be required to assist educators in developing relevant data interrogation skills and to acknowledge the usefulness of data in their practice.

If a population health approach were to be applied to educational planning, with the ultimate goal of supporting children’s development, it would require consideration of the supports and structures already in place at both the local and systemic levels. Recognising the differing goals of health and education systems, any approaches applied would need to be modified for the environment and goals of the education sector. It is likely that a new approach, specifically designed with education at the helm, would be required to meet the needs and restraints of the system. An ‘education promotion approach’ could see improved stakeholder relationships prior to school entry and ultimately improved outcomes for children.

## Conclusion

This review utilised the CIS method to outline the key concepts that occurred in the literature around population health approaches and their application to education planning and children’s early development. Within education, there are a number of models which are used to improve outcomes for children and families. Although a population health approach to planning does not explicitly exist within education, the results from this review indicate that it would indeed be possible to adapt the population health approach to prior-to-school and school educational planning, and that doing so is likely to be advantageous for children’s development. Presently, there is a dearth of research demonstrating this benefit, and more work is needed to articulate the ways in which population data adds value to schools, and the extent to which this type of planning improves the experiences of children in school and their educational outcomes. Finally, implementing such an approach will require system changes and supports that enable schools to connect with their communities and flexibility to respond to the context of children.

Thus, this review asserts several key questions that could guide future research or inform practice. Firstly, does population data enhance educator understanding of context and factors driving children’s learning and development, and in this way planning for children’s development and learning? Secondly, how can partnerships support educators to plan holistically from a population-based perspective? Finally, are schools able to work with communities prior to children entering school and if so, what impact does this have on children’s development at school entry?

## Supporting information

S1 File Data extraction table. (DOCX)

S2 File PRISMA checklist. (PDF)
