# Supplementary material for: A population health approach in education to support children’s early development: A Critical Interpretive Synthesis
Source: PLoS One. 2019 Jun 14;14(6):e0218403. doi: 10.1371/journal.pone.0218403 (PMC6568401; doi:10.1371/journal.pone.0218403)
Supplement: S1 File — (DOCX) [file pone.0218403.s001.docx]

# **Data extraction table**

| **Reference** | **Title** | **Type of paper** | **Methods** | **Summary** | **Key Findings** | **Concepts** |
| --- | --- | --- | --- | --- | --- | --- |
| *Adelman & Taylor (2015)* | Whole Child Schooling and the 2015 National Initiative for Transforming Student and Learning Supports | Commentary | Discussion of current approaches to student learning and supports | Describes the fragmented nature of school planning and promotes the need for collaborative approaches to improve student and learning supports. | Learning and student supports differ across schools, though are generally fragmented. This fundamental policy problem leads to schools ineffectively addressing barriers to student and learning supports, duplication across services and marginalisation. Interrelated solutions require collaboration and sharing of resources, connecting schools with families and communities to achieve a common goal. | School planning, community collaboration |
| *Ambrose & Short (2009)* | Integrating health planning and social planning: a case study in community-based partnerships for better health | Primary research | Case study. Interviews and document reviews | The study investigated the process by which health and social planning were integrated in practice at the community level. A retrospective study of a community-based health promotion project for early childhood environments was conducted. | Planning followed a similar process as the 'Health Promoting Schools' model, but was also influenced by local, state and federal policy, as well as emerging research about the importance of early childhood development and health. Time constraints were discussed as a significant barrier in planning and linking to other community services/agencies. Professional development, networking and partnerships and inclusion, engagement and change all lead to an increase in empowerment in staff. | Collaboration, stakeholder partnerships, increased engagement, program planning |
| *Amed (2015)* | Creating a collective impact on childhood obesity: Lessons from the SCOPE initiative | Primary research | Community-based participatory research | Describes the processes used in SCOPE to achieve multi-sectorial engagement and collective action to prevent childhood obesity | A multi-pronged community-led childhood obesity prevention initiative can be achieved by using community-based participatory research principles. Best practice processes were found to align with the principles of CBPR and the conditions of collective impact. | Community-based participatory research, collective impact, community engagement |
| *Bassett-Gunter et al. (2016)* | Health school communities in Canada | Commentary | Review of key resources to identify common components and principles necessary for a healthy school communities approach | Describes the process for enhancing communication among organisations and stakeholders invested in health school communities internationally | Education, social and physical environments, policy, community partnerships and the use of evidence were all identified as core components of healthy school communities. The adoption of a whole school approach, education and health service synergy, planning and assessment, leadership and sustainability were discussed as fundamental principles. | School planning, healthy school communities, school health and well-being |
| *Belansky et al. (2011)* | An Adapted Version of Intervention Mapping (AIM) Is a tool for conducting community-based participatory research | Primary research | Community-based participatory research, Intervention mapping and interviews | The adapted version of Intervention Mapping is closely aligned to 7 of the 9 community-based participatory research principals | A strong appreciation for outside facilitation of the planning process and resources were key themes emerging from the analysis. The value of research partners was discussed in terms of sharing data and success stories, evidence-based practices and administrative support (organisation of meetings). Outside facilitation was viewed positively, as it allowed members to feel they had an equal vote in the discussion. | Collaboration, intervention-mapping, school planning, community-academic partnerships |
| *Blank (2015)* | Building sustainable health and education partnerships: stories from local communities | Primary research | Case study. Interviews and document reviews | This paper describes community schools, their relationships with partners and the outcomes associated with sustainable partnerships | Schools and districts with strong health partnerships reflecting community schools strategy have been shown to improve attendance, academic performance and increase access to mental, dental, vision and health supports for their students. Leadership and management infrastructure was discussed as necessary for success. Data, professional development and a focus on results all support outcomes | School planning, cross-sector patronships, education and health outcomes, data |
| *Bolton et al. (2017)* | The outcomes of health-promoting communities: being active eating well initiative - a community-based obesity prevention intervention in Victoria, Australia | Primary research | Mixed method, multilevel quasi-experimental evaluation | This study evaluates the impact of the Health-Promoting Communities: Being Active Eating Well initiative. | Common strategies included social marketing, stakeholder engagement, network and partnership development, community-directed needs assessment and capacity building. Gains were shown in community capacity. Results varied across communities, with one achieving a higher level of healthy eating policy in schools, two with improved healthy eating related behaviours, one with lower sedentary behaviours and one with higher levels of physical activity in schools. High-level multi-sectoral partnerships across government departments and state-level policies in education settings are recommended. It is suggested that future research should consider a systems approach modified existing systems rather than relying on the addition of new activities. To achieve effective and sustainable solutions, strategies need to become integrated into existing practice and operating systems to support the community in general. | Health promotion, healthy communities, intervention, policy |
| *Bostock (2018)* | Diffusion theory and multi-disciplinary working in children's services | Primary research | Qualitative. Interviews | Explores how innovation in children's services is adopted and developed by staff within new multi-disciplinary teams. | DOI theory is described, identifying the five innovation attributes that are important for rapid diffusion: rapid advantage compatibility, complexity, trialability and observability. Multi-disciplinary team working and group supervision were viewed as advantageous and improved the service to children and families. DOI was viewed as a useful reflective tool for senior managers to plan and review change programmes and to identify and emerging barriers to successful implementation | Diffusion of innovations, multi-disciplinary planning |
| *Bruce, Klein, Keleher (2012)* | Parliamentary inquiry into health promoting schools in Victoria: Analysis of Stakeholder views | Primary research | Thematic analysis of parliamentary submissions | This paper discusses the barriers and enablers to school health promotion, including the need for stronger leadership from the Departments of Health and Education and Early Childhood Development. | Submissions (from the Inquiry) supposed the need for increased resources allocation to support health promotion in schools, and for coordinated approached with stronger leadership from the health and education sectors, rather than supporting the idea that schools could have a wider role in communities. This structure would allow schools to address health in a more strategic manner, with increased resources, capacity and outcomes. | Health promoting schools, school health |
| *Can (2015)* | The value of using schools and community assets for health | Review | Meta-synthesis | This review investigates if the wider learning environment provided in a school is valuable in improving health. | Asset-based Community Development was described as a useful lens to view research in schools on the interaction of education and health improvement. This model was discussed as potentially useful for national governments to develop resources for education and health. | School planning, community development, community assets, collaboration |
| *Campo (2017)* | Examining school leadership in New York Community schools | Primary research | Interviews, surveys | The community school model is rooted in John Dewey's (1902) conceptualization of the public school as a hub for the community. This work has evolved over a hundred years and recently experienced prominence in the public eye as a fundamental component of New York City's school turnaround policy. This dissertation describes findings and recommendations from interviews with leaders in ten New York City community schools. These interviews are triangulated with analysis of the New York City school environment survey using both faculty and parent responses. This research investigated the values, processes and behaviours of leaders that hinder or contribute to the partnership between community-based organizations (CBO) and the Department of Education (DOE). | Principals of community schools must undergo a paradigm shift, shaped by the understanding that leading a community school is different from a traditional public school, and requires specific skills and behaviours. Primarily, school leaders need to cultivate a distributed leadership approach supported by practices of mindfulness and the cultivation of collaborative systems. In order to create a healthy school climate, they must develop processes to collaboratively create school values and vision and periodically revisit how these values are being supported by the community school. This work is hindered by a lack of specific policies supporting community school management and daily practices, as well as an unclear definition of roles and hierarchy. An emerging theme was the disproportional cultural capital of CBO staff compared to DOE staff, and the author provides recommendations to address the accountability imbalance which reinforces this dynamic between the two factions. | Community schools, teacher leadership |
| *Cappella (2008)* | Enhancing schools' capacity to support children in poverty: an ecological model of school-based mental health services | Commentary | Discussion on refocusing school-based services in poor communities on the core function of schools to promote learning. | An ecological model informed by public health and organisational theories to refocus school-based mental health services in high poverty communities on the core function of schools to promote learning is proposed. The influence of poverty on child development and the limitations of current models of prevention and intervention in schools is also discussed. | Schools are discussed as having an inherent capacity to support children's mental health and development, and to bridge critical home and community ecologies. Underfunded schools do not have the adequate environments to protect children from the influence of risk. Ecological, public health and organisational theories can be used to reconceptualise the service delivery in these communities to focus on schools' core function and inherent capacity to promote children's learning and development. Influential individuals from among the school or community were also mentioned as able to enhance the effectiveness and sustainability of services and help bridge the gap between university-based research and community practices. | Ecological model, organisational theory, school planning, collaboration |
| *Close (2012)* | Developing multi-agency leadership in education | Review | Review of multi-agency leadership | This article contributes to the knowledge of how multi-agency leadership in children and young people's services is understood and developed. It presents a framework for multi-agency leadership development. | The framework presented comprises six elements: complexity and culture, multi-professional groups and inter-organisational partnerships and organisational consultancy and professional supervision. Ethical maturity must be achieved if multi-agency leadership is to benefit children and young people. | Leadership, educational change, inter-disciplinary research |
| *Comer & Ben-Avie (2010)* | Promoting community in early childhood programs: a comparison of two programs | Primary research | Cross-sectional | Describes the Jewish Early Childhood Education Initiative, exploring quality in early childhood education, the impact of early childhood programs on the lifepaths of families, and the contribution of the families to the programs | JECEI implemented a governance and management process that improved relationships and increased the capacity of early childhood centres to impact the families. A governance and management process that enables the school community to anticipate and manage change, as well as coordinator all initiative is required to successful engagement. JECEI families were positively influences by the early childhood program, resulting in better connected communities and families. | Early childhood education programs, family engagement, school improvement |
| *Corter & Pelletier (2015)* | Schools as Integrated Service Hubs for Young Children and Families: Policy Implications of the Toronto First Duty Project | Commentary | Describes policy implications of Toronto First Duty in Canada | The paper describes the Toronto First Duty Project, designed to test the feasibility and effects of a universal model for integrating child care, kindergarten, families support and other services in school-based community hubs. | There was positive evidence on the feasibility of implementing the model, as well as evidence about the processes that work through program and family pathways to enhance child development and parenting. Positive outcomes for children's development were presented, alongside improved quality of family life. Experience from the Toronto First Duty project suggests that ongoing research and development will be an important part of ensuring the success of system change. | integrated services, policy |
| *Denman (1999)* | Health promoting schools in England - a way forward in development | Commentary | Describes health promoting schools in England | The paper reviews research and practice and makes recommendations to inform the future development of schools and health promoting organisations. | The model is described as process driven, requiring commitment and an ability to adapt to the changing needs of the school community. External agencies can stimulate and support schools in their endeavours. | Health promoting schools, school health |
| *Dworkin & Sood (2016)* | A population health approach to system transformation for children's healthy development | Commentary | Describes the Help Me Grow program alongside the implications from the science of early brain/child development and adversity and toxic stress. | This article shares the story of Help Me Grow, a program designed to support children across a range of developmental trajectories, linking children and their families to community-based programs and services to strengthen protective factors that promote children's optimal healthy development. | Cross-sector collaboration is essential to address families' needs. Focusing only on children with complex needs fails to acknowledge the benefits of targeting the needs of all children in a population health approach. System building should encompass all sectors that influence family’s capacity and well-being. | Population health approach, social determinants, child development |
| *Ellis & Dietz (2017)* | A new framework for addressing adverse childhood and community experiences: the building community resilience model | Primary research | Qualitative. Interviews and focus groups | Proposes a transformative approach to foster collaboration across agencies to address the causes of toxic stress and childhood adversity to build community resilience. | The Building Community Resilience model as a means to explore capacity issues, reduce fragmented health care deliver and facilitate integrated systems across partners was supported. | Adverse childhood experiences, integrated models, community resilience |
| *Gleddie (2010)* | A journey into school health promotion: direct implementation of the health promoting schools’ approach | Primary research | Case study. Interviews, focus groups, document analysis, observations | Describes the Battle River Project in line with the health-promoting schools’ approach. The original study aimed to examine the effectiveness of a local school district implementation model of the HPS. | Participation, coordination and integration were identified as descriptors to how the program worked and was organised. The program was particularly effective in stimulating the development of policy and programs grounded in the Health Promoting Schools framework, inclusive of physical activity, healthy eating and mental well-being. A systemic divisional approach was discussed as a successful approach that considers local differences and is an effective use of resources. | Health promoting schools |
| *Haggett (2002)* | School-community engagement: an opportunity for relational leadership theory in action | Primary research | Multi-method study, interviews, observation, surveys | Explores systems theory, change theory and relational theory and how the three connect to guide the research in an enactment of a leadership opportunity a forum for school=community dialogues on best practices in education. | Educational leadership can relate a climate that ignites a leadership community, rather than relying on a hero-leader. This paper also provides examples of how educators and the public can cultivate healthy relationships that support beneficial change. | Leadership, community collaboration, schools, engagement |
| *Halfon et al. (2004)* | Building bridges: A comprehensive system for healthy development and school readiness | Report | Discussion of the State Early Childhood Comprehensive Systems Initiative: a policy designed to improve early childhood programs to ensure that all children arrive at school health and ready to learn. | This report focuses on bridging concepts, platforms, pathways, strategies, and tools that can be used by SECCS grantees to achieve the goals of the initiative. | Strategies for the engagement process are described as well as how to create a common framework for systems building | Collaboration, planning |
| *Hayman (2014)* | Personal, social, health and economic education: the bridge between public health and education | Commentary | Discusses how schools can achieve public health objectives and develop partnerships | Describes the Healthy School approach and explores ways in which schools and local authorities can work together to achieve shared outcomes. | The healthy school approach can bridge the gap between positive health outcomes and educational attainment. Local authority investment was discussed as providing support to schools. The approach is suggested to have significant impact on both school and local authority priorities, enhancing health and well-being and supporting academic achievement | Healthy school communities, collaboration |
| *Healthy Communities, Healthy Kids (2009)* | Healthy Communities, Healthy Kids: How Ophea's Living Schools are pointing the way to a healthier lifestyle for Canada's kids | Commentary | Discusses Ophea's Living Schools | The common process of Living Schools is presented, alongside the six critical success factors for creating healthy schools. | Living School approach recognises the factors contributing to poor health outcomes. The concept is a lens through which a school's existing activities can be viewed and enhanced. Pillar partners (students, parents, local businesses and other community leaders) are discussed as necessary for developing and implementing plans. Critical success factors are discussed: shared leadership and responsibility, a clear and common vision, clearly articulated process steps, dedicated and sustained funding and support, assessment, monitoring and evaluation and establishment of a healthy school’s office or department. Living schools saw a number of positive outcomes through test score, behaviour, belonging, physical health and attendance. | Partnerships, school health, child development |
| *Hopson & Lawson (2011)* | Social workers' leadership for positive school climates via data-informed planning and decision making | Primary research | Cross-sectional | The paper discusses the role of social workers in schools, developing a schoolwide understanding of students need and resources and the potential for leadership roles. The importance of data-informed decision making is discussed. | A positive school climate requires data-informed decision making. Data can also be applied to district improvement plans. A data-informed theoretical framework for developing a positive school climate by providing opportunities, skills and reinforcement is proposed. | Academic outcomes, evidence-based practice |
| *Horn, Freeland, Butler (2015)* | Schools as Community Hubs: Integrating support services to drive educational outcomes | Commentary | Discusses schools as community hubs | Discusses how schools can function in an interdependent manner with other service providers yet maintain the control needed to customise services to student's needs and achieve academic outcomes | Student information sharing was discussed as often difficult but to privacy rules. The impact of hub-based services in the wider community was mentioned as rarely measured fully or reflected in budgets for the hub. Authors recommend a wider use of budget waivers and specialised training for school leaders to coordinate services efficiently. | School-based hubs, integrated services, policy |
| *Israel (1998)* | Review of community-based research: Assessing partnership approaches to improve public health | Review | Discussion of the literature on community-based and related forms of research | Provides a synthesis of key principles of community-based research, examines its place within the context of different scientific paradigms, discusses rationales for its use, and explores major challenges and facilitating factors and their implications for conducting effective community-based research aimed at improving the public's health | Key principles can be summarised as: recognises community as a unit of identity; builds on strengths and resources within the community; facilitates collaborative partnerships in all phases of the research; integrates knowledge and action for mutual benefit of all partners, promotes a co-learning and empowering process that attends to social inequalities, involves a cyclical and iterative process, addresses health from both positive and ecological perspectives and disseminates findings and knowledge gained to all partners. Challenges associated with the approach include partnership related issues such as a lack of trust and respect, methodological issues such as proving intervention success, and broader social, political, economic, institutional and cultural issues. | Community-centred, collaborative, public health partnerships |
| *Iversen (2006)* | Developing a participatory multidisciplinary team approach to enhance the quality of school start | Primary research | Participatory action research | Discusses how PAR was applied at 27 schools in Norway, to enhance the quality of school start | Multidisciplinary teamwork and relationships were improved and there was an increased focus on developmental and health care issues. Authors also report that professional knowledge and practical skills and support to local educational staff was improved. Local creativity and ownership with supportive administrative structures were discussed as promoting factors. Time and external professional resources were reported as the main barriers. | Multi-disciplinary teamwork, Children's development |
| *Langford et al. (2014)* | The WHO health promoting school framework for improving health and well-being of students and their academic achievement (review) | Review | Systematic | A systematic review assessing the effectiveness of the Health Promoting Schools framework in improving the health and well-being of students and their academic achievement | Positive effects for some interventions for: FMI, physical activity, fitness, fruit and vegetable intake, tobacco use and being bullied were found. Few studies included any academic, attendance or school-related outcomes. | Health promoting schools, outcomes |
| *Lewallen (2015)* | The Whole School, Whole Community, Whole Child Model: A new Approach for Improving Educational Attainment and Healthy Development of Students | Review | Discusses the development of the Whole School, Whole Community, Whole Child approach. | Experts from education and health discussed lessons learned from the implementation of the coordinated school health and Whole child approaches. As a result of the discussions the Whole School, Whole Community, Whole Child approach was developed. | The WSCC approach provides opportunities that will improve education attainment and healthy development for students. Community engagement, policies, processes and practices build upon the strengths of each approach and address the needs of modern schools. | Coordinated school health, whole child approach |
| *Lowe et al. (2001)* | School-based health centers as a locus for Community Health Improvement | Commentary | Discussion of school-based health centres | This paper discusses how health professionals and educators can collaborate effectively in addressing the specific concerns of school attendance and teen smoking. | School-based health centres were found to be critical resources for providing and coordinating services (health and medical) for children and adolescents. | School-based hubs, integrated services |
| *Mort (2007)* | School districts in Community Intersectoral Coalitions: models of collaboration for young children | Primary research | Mixed-methods. Focus groups, interviews, observations. | This dissertation explored how school districts participated in successful interdisciplinary community coalitions, to improve the quality of and the opportunities for services of young children and their families, resulting in school success. | The coalitions work resulted in improved coordination, services and access to programs for the early learning of young children. Schools and districts were discussed as playing a key role, the need for reliable data was also described, as well as the need for sustainable and transformative leadership that is able to evolve. Government support is required for grassroots movements by new service reorganisation, funding mechanisms and related policy redevelopment. | Interdisciplinary, children's development, collaboration |
| *Murray et al. (2007)* | Coordinated school health programs and academic achievement: a systematic review of the literature | Review | Systematic | This review examines the evidence that school health programs aligned with the Coordinated School Health Program model improve academic success | A positive effect was found on some academic outcomes from school health programs for asthmatic children. A lack of evidence exists for negative effects of physical education programs on academic outcomes. Limited evidence exists to support the effect of health, nutrition and mental health services. No evidence was found supported the effect of staff health promotion programs or school environment interventions on academic outcomes. | Academic achievement, coordinated school health |
| *Murray (2015)* | Supporting the whole child through coordinated policies, processes and practices | Commentary | Discusses the roles of the school district and of schools in creating optimal learning environments that support the whole child. | This paper discusses the WSCC model, determining key factors to success. | Three factors led to successfully implementing WCSS: hiring a coordinator at the district and school levels, having collaborative teams address health and learning at the district and school levels, and using data to make sessions and build health outcomes into accountability systems. | Whole child model, school health, community collaboration |
| *Poduska (2012)* | Developing a collaboration with the Houston Independent School District: Testing the generalizability of a partnership model | Commentary | Discusses the development of a academic/school partnership | This paper describes the development of a partnership between the Houston Independent School District, The Houston Federation of Teachers and the American Institutes of Research, aiming looking at the Good Behaviour Game. | The partnership was summarised in a six-step framework: analyse the social/political context; learn abut the problems, priorities, and vision of community leaders; identify mutual self-interest; develop a common vision; request ad hoc oversight from district and community leaders; work through issues of trust. | Researcher-practitioner partnerships, community-based participatory research |
| *Rooney* | Using the whole school, whole community, whole child model: Implications for practice | Commentary | Discusses the Whole School, Whole community, Whole Child model | This article discusses the strategies, steps and resources that can be integrated into existing process, that help to improve health and academic outcomes. | Administrative support was identified as an important factor for implementing strategies and sustaining efforts. Other key elements of the planning phase include strategy selection and evaluation, which guides successes and provides direction for program adjustment, | Whole child model, school health, community collaboration |
| *Schools (2005)* | Schools: The Perfect place to address the needs of the whole child' | Commentary | Discusses the role of schools in addressing the needs of the whole child | This article describes the role that schools can play as institutions, bringing the community into schools and acting as a service point for youth development and support. | Schools can have a significant impact on children's lives, therefore engagement is crucial for improved outcomes. Authors recommend schools adopt a Whole-Child approach that incorporates roles for families and communities, improve school climate and increase student connectedness, align resources with needs, align goals across agencies and provide incentives to reward improved coordination and fight for local, state and federal community school initiatives. | School-based hubs, integrated services, policy |
| *Slade & Griffith (2013)* | A whole child approach to student success | Commentary | Discusses the Whole Child approach | The conception of the Whole Child Approach is discussed as well as the policy and economic implications | Improvement strategies should be evidence based. Educators need help to support students through funding and training. Ensuring that all children are healthy, safe, engaged, supported and challenged should be a national priority. Parents, educators and community members should be engaged to provide a whole child approach to education. | Whole child model, school improvement |
| *St Leger (1999)* | The opportunities and effectiveness of the health promoting primary school in improving child health - a review of the claims and evidence | Review | Review of the opportunities and effectiveness of the health promoting primary school in improving child health | This article disuses the health promoting school and the claims and evidence from the school health research literature, focused on primary schools | Health gains for primary school students are most likely to occur if a well-designed program is implemented which links the curriculum with other health promoting school actions, contains professional development and is underlined by a theoretical model. | Child health, health promoting school |
| *Thomas, Rowe & Harris (2010)* | Understanding the factors that characterize school-community partnerships The case of the Logan Healthy Schools Project | Primary research | Qualitative. Interviews and observations | This study examines the factors that make up an effective school-community partnership that support the sustainability of school health initiatives applied within a health-promoting schools approach | A focus on building relationships between school and community partners, complementary capacities, commonality of intent and shared goals, competence of practice were all described as key components of success. | Health promoting schools, partnerships, collaboration |
| *Valois et al. (2015)* | The ASCD Health School Communities project formative evaluation results | Primary research | Evaluation including qualitative component (site visits, interviews), document analysis, report card results and school improvement plans | This evaluation utilised 11 sites to determine the levels of change in a school community that allow for the initiation and implementation of best practice and policy for improving school health | Nine key elements were determine: principal as leader of the efforts, active and engaged leadership, distributive team leadership, effective use of data, integration of the process with the school improvement process, ongoing and embedded PD, authentic and mutually beneficial community collaborations, stakeholder support of the effect, creation or modification of policy | Health promoting schools, outcomes, healthy school community |
| *Weist, Ambrose & Lewis (2006)* | Expanded school mental health: a collaborative community-school example | Commentary | Discusses a mental health framework involving school and community staff | This article discuses a school mental health framework that through the involvement of school and community staff enhances mental health programs for youths | Working across disciplines and constraints of job duties were described as challenges to collaboration. Sustaining school-community program collaboration was discussed in term of funding and investment of energy in building a collaborative relationship. Support from leadership was crucial in overcoming this barrier. Communication, decision making and mutual support were discussed, Positive channels of communication need to be established early, gaps in services and unmet needs need to be addressed and feedback should be sought. | Collaboration |
| *Whitcomb (2009)* | Strong start: Impact of direct teaching of a social-emotional learning curriculum and infusion of skills on emotion knowledge of first grade students | Primary research | Quasi-experimental | This dissertation discusses a pilot study that examined the impact of the program on first grade students social-emotional knowledge skills. | Students’ knowledge about emotional situations was increased, while students internalising behaviours decreased with exposure to the program. | Social-emotional, population health approach, mental health |

# **Synthetic construct analysis**

| Synthetic Construct | General theme | Topics included | References |
| --- | --- | --- | --- |
| **Collaboration and partnerships possess unique opportunities to influence children's development** | Collaboration & partnerships to support children's development | Collaboration | Ambrose & Short (2009) |
|  |  |  | Belansky et al. (2011) |
|  |  |  | Can (2015) |
|  |  |  | Halfon et al. (2004) |
|  |  |  | Hayman (2014) |
|  |  |  | Israel (1998) |
|  |  |  | Thomas, Rowe & Harris (2010) |
|  |  |  | Weist, Ambrose & Lewis (2006) |
|  |  | Collective impact | Amed (2015) |
|  |  | Community-academic partnerships | Belansky et al. (2011) |
|  |  | Community assets | Can (2015) |
|  |  | Community-based participatory research | Amed (2015) |
|  |  |  | Poduska (2012) |
|  |  | Community collaboration | Adelman & Taylor (2015) |
|  |  |  | Cappella (2008) |
|  |  |  | Haggett (2002) |
|  |  |  | Mort (2007) |
|  |  |  | Murray (2015) |
|  |  |  | Rooney |
|  |  | Community development | Can (2015) |
|  |  | Cross-sector partnership's | Blank (2015) |
|  |  | Data | Blank (2015) |
|  |  | Interdisciplinary | Close (2012) |
|  |  |  | Mort (2007) |
|  |  | Multi-disciplinary planning | Bostock (2018) |
|  |  |  | Iversen (2006) |
|  |  | Multi-disciplinary teamwork | Iversen (2006) |
|  |  | Organizational theory | Cappella (2008) |
|  |  | Partnerships | Ambrose & Short (2009) |
|  |  |  | Healthy Communities, Healthy Kids (2009) |
|  |  |  | Thomas, Rowe & Harris (2010) |
|  |  | Policy | Bolton et al. (2017) |
|  |  |  | Corter & Pelletier (2015) |
|  |  | Public health partnerships | Israel (1998) |
|  |  | Researcher-practitioner partnerships | Poduska (2012) |
|  |  | Stakeholder partnerships | Ambrose & Short (2009) |
| **Children's development can be influenced at a variety of levels** | Factors influencing children's development | Adverse childhood experiences | Ellis & Dietz (2017) |
|  |  | Children's development | Dworkin & Sood (2016) |
|  |  |  | Iversen (2006) |
|  |  |  | Mort (2007) |
|  |  |  | St Leger (1999) |
|  |  | Community resilience | Ellis & Dietz (2017) |
|  |  | Early childhood education programs | Comer & Ben-Avie (2010) |
|  |  | Family engagement | Comer & Ben-Avie (2010) |
|  |  | Intervention | Bolton et al. (2017) |
|  |  | Intervention-mapping | Belansky et al. (2011) |
|  |  | Mental health | Whitcomb (2009) |
|  |  | Social determinants | Dworkin & Sood (2016) |
|  | Outcomes | Academic achievement | Murray et al. (2007) |
|  |  | Academic outcomes | Hopson & Lawson (2011) |
|  |  | education and health outcomes | Blank (2015) |
|  |  | Social-emotional | Whitcomb (2009) |
|  |  | Outcomes | Langford et al. (2014) |
|  |  |  | Valois et al. (2015) |
| **Population health models exist within public health and can help improve outcomes for more children** | Population health models | Community-centered | Israel (1998) |
|  |  | coordinated school health | Murray et al. (2007) |
|  |  |  | Lewallen (2015) |
|  |  | Ecological model | Cappella (2008) |
|  |  | Evidence-based practice | Hopson & Lawson (2011) |
|  |  | Healthy communities | Bassett-Gunter et al. (2016) |
|  |  |  | Bolton et al. (2017) |
|  |  | Health promotion | Bolton et al. (2017) |
|  |  |  | Valois et al. (2015) |
|  |  | Health promoting schools | Bruce, Klein, Keleher (2012) |
|  |  |  | Denman (1999) |
|  |  |  | Gleddie (2010) |
|  |  |  | Langford et al. (2014) |
|  |  |  | St Leger (1999) |
|  |  |  | Thomas, Rowe & Harris (2010) |
|  |  | Healthy school communities | Hayman (2014) |
|  |  |  | Valois et al. (2015) |
|  |  | Integrated models | Corter & Pelletier (2015) |
|  |  |  | Ellis & Dietz (2017) |
|  |  | Integrated services | Lowe et al. (2001) |
|  |  |  | Schools (2005) |
|  |  | Population health approach | Dworkin & Sood (2016) |
|  |  |  | Whitcomb (2009) |
|  |  | Whole child approach | Lewallen (2015) |
|  |  |  | Murray (2015) |
|  |  |  | Rooney |
|  |  |  | Slade & Griffith (2013) |
| **System change requires a range of drivers and supports** | Supporting development in education settings | Diffusion of innovations | Bostock (2018) |
|  |  | Educational change | Close (2012) |
|  |  | Increased engagement | Ambrose & Short (2009) |
|  |  |  | Haggett (2002) |
|  |  | Leadership | Close (2012) |
|  |  |  | Haggett (2002) |
|  |  | Program planning | Ambrose & Short (2009) |
|  |  | School-based hubs | Lowe et al. (2001) |
|  |  |  | Schools (2005) |
|  |  | School health | Bruce, Klein, Keleher (2012) |
|  |  |  | Denman (1999) |
|  |  |  | Healthy Communities, Healthy Kids (2009) |
|  |  |  | Murray (2015) |
|  |  |  | Rooney |
|  |  | School health and well-being | Bassett-Gunter et al. (2016) |
|  |  | School improvement | Comer & Ben-Avie (2010) |
|  |  |  | Slade & Griffith (2013) |
|  |  | School planning | Adelman & Taylor (2015) |
|  |  |  | Bassett-Gunter et al. (2016) |
|  |  |  | Belansky et al. (2011) |
|  |  |  | Blank (2015) |
|  |  |  | Can (2015) |
|  |  |  | Cappella (2008) |
|  |  |  | Halfon et al. (2004) |
